# Effects of a Novel, Intelligent, pH-Responsive Resin Adhesive on Cariogenic Biofilms In Vitro

**DOI:** 10.3390/pathogens11091014

**Published:** 2022-09-05

**Authors:** Yangyang Shi, Jingou Liang, Xuedong Zhou, Biao Ren, Haohao Wang, Qi Han, Hao Li, Lei Cheng

**Affiliations:** 1 State Key Laboratory of Oral Diseases, West China Hospital of Stomatology, National Clinical Research Center for Oral Diseases, Sichuan University, Chengdu 610041, China; 2 Department of Operative Dentistry and Endodontics, West China School of Stomatology, Sichuan University, Chengdu 610041, China; 3 Department of Pediatric Dentistry, West China School of Stomatology, Sichuan University, Chengdu 610041, China; 4 Department of Oral Pathology, West China School of Stomatology, Sichuan University, Chengdu 610041, China

**Keywords:** *Streptococcus mutans*, *Candida albicans*, secondary caries, antimicrobial effect, pH response

## Abstract

Background: Secondary caries often result in a high failure rate of resin composite restoration. Herein, we studied the dodecylmethylaminoethyl methacrylate–modified resin adhesive (DMAEM@RA) to investigate its pH-responsive antimicrobial effect on *Streptococcus mutans* and *Candida albicans* dual-species biofilms and on secondary caries. Methods: Firstly, the pH-responsive antimicrobial experiments including colony-forming units, scanning electron microscopy and exopoly-saccharide staining were measured. Secondly, lactic acid measurement and transverse microradiography analysis were performed to determine the preventive effect of DMAEM@RA on secondary caries. Lastly, quantitative real-time PCR was applied to investigate the antimicrobial effect of DMAEM@RA on cariogenic virulence genes. Results: DMAEM@RA significantly inhibited the growth, EPS, and acid production of *Streptococcus mutans* and *Candida albicans* dual-species biofilms under acidic environments (*p* < 0.05). Moreover, at pH 5 and 5.5, DMAEM@RA remarkably decreased the mineral loss and lesion depth of tooth hard tissue (*p* < 0.05) and down-regulated the expression of cariogenic genes, virulence-associated genes, and pH-regulated genes of dual-species biofilms (*p* < 0.05). Conclusions: DMAEM@RA played an antibiofilm role on *Streptococcus mutans* and *Candida albicans* dual-species biofilms, prevented the demineralization process, and attenuated cariogenic virulence in a pH-dependent manner.

## 1. Introduction

Dental caries are the most common chronic bacterial infectious oral diseases, and their high incidence rate and wide range can seriously endanger people’s oral health and even general health, causing considerable economic and quality of life burdens [1,2]. A study published in 2017 shows that the prevalence of permanent tooth caries ranks first among 328 diseases, the incidence rate ranks second, and the incidence rate of deciduous tooth caries ranks fifth [3]. 

ECC, short for early childhood caries, are special kinds of dental caries affecting children under 6 years old [4,5], and they have a high prevalence all over the world. An analysis of 72 global studies from 1998 to 2018 found that the average prevalence of caries in 1-year-old children was approximately 17%, and the prevalence of dental caries in 2-year-olds increased significantly to 36%. Meanwhile, the prevalence of caries in children aged 3, 4, and 5 were 43%, 55%, and 63%, respectively [6]. In China, the Fourth National Oral Health Survey showed that the prevalence of caries in 5-year-olds was 70.9%, and this figure had increased by 5.8% compared to 10 years earlier, which means the incidence of ECC in China has increased over the past decade [7]. It is obvious that ECC significantly affects children’s quality of life and represents great challenges for the public health system due to its negative consequences, such as infections, chronic pain, obstructing diet and chewing, sleeping disorders, and tooth extraction, all of which affect both oral and general health [8]. Therefore, the prevention and treatment of early childhood caries in young children has aroused widespread concern.

So far, resin composite restoration is the most common treatment for dental caries [9]. However, at present, resin composite restoration still has a considerably high failure rate and may affect a patient’s long-term prognosis. Relevant clinical retrospective studies have found that the failure rate of resin composite restorations can reach 50%, among which the occurrence of secondary caries was one of the main causes [10,11,12]. For children with ECC, due to the low mineralization of deciduous teeth, poor oral health maintenance, limited cooperation during restoration treatments, and other reasons, the incidence of secondary caries is higher than it is for adults. 

The development of secondary caries is a complex process which combines the well-known etiology of “conventional” caries and the particularity of restoration materials. At present, a general belief is that the cariogenic biofilm of secondary caries is similar or the same as that of primary caries [13]. A growing number of studies support that *Streptococcus mutans* (*S. mutans*) and *Candida albicans* (*C. albicans*) dual-species biofilms play an important role in the formation of ECC [14,15]. *S. mutans* is known to produce acid and tolerate low pH values, which further causes tooth surface demineralization and the formation of dental caries [16]. Further, the virulence properties of *C. albicans*, such as its acidogenicity and aciduric nature, ability to form rich biofilms, fermentation and assimilation of dietary sugar, and production of collagen soluble proteases all indicate that *C. albicans* has a latent cariogenic potential [17]. Furthermore, existing studies indicate that the presence of *C. albicans* is very important in maintaining an acidic environment with *S. mutans* and enhancing the maturity of dual-species biofilms, which means that the dual-species collaboration may lead to a vicious circle and is conducive to enamel demineralization, which aggravates the severity of dental caries and the possibility of filling failures in young children [18,19,20,21,22]. Therefore, to prevent secondary caries in ECC, paying attention to the antimicrobial effect of this dual species, especially in acidic environment, is very important.

On the other hand, the properties of restoration materials themselves are also closely related to the occurrence of secondary caries. Many attempts have been made to improve the antimicrobial activity of restorative materials. Intraoral pH is an important factor affecting the occurrence of caries. Under physiological conditions, intraoral pH is maintained at neutral, while according to Stephan Curve, after sugar exposure, the local pH of dental plaque can be reduced to 5, which is lower than critical pH (a pH of 5.5) for caries development [23]. However, most of the current studies have focused on the direct analysis of their antimicrobial ability, ignoring their ability to act in a cariogenic acidic environment. This means that in acidic environment, the antimicrobial effect of some materials may deteriorate [24] and the occurrence of secondary caries cannot be effectively reduced.

Hence, correspondingly, intelligent pH responsive agents and materials that only play an antimicrobial effect at a low pH are exactly right for being applied to the unique acidic environment of dental caries, and they have attracted great attention in antimicrobial research to prevent secondary caries [25]. There have been a few reports that indicated that antimicrobial agents such as farnesol [26], doxycycline [27], and chlorhexidine [25] were loaded on pH-activated nanocarriers to control drug release. However, these drug release systems usually showed only temporary effects because once the loaded antibiotics were released, they were difficult to recharge. 

As described in our previous studies, we have synthesized a novel pH-responsive material, dodecylmethylaminoethyl methacrylate (DMAEM), which can be protonated under acidic conditions and play an antimicrobial role in preventing dental caries. Our previous study demonstrated that DMAEM-modified resin adhesive (DMAEM@RA), by adding 5% DMAEM, could provide the long-term inhibition of *S. mutans* without affecting the mechanical properties and biocompatibility of dental adhesive system [28]. 

Moreover, DMAEM@RA is different from traditional drug release systems, with no separate drug carrier, and can repeatedly respond to pH changes in vivo and exert an antimicrobial activity. Therefore, DMAEM@RA may have the potential to prevent secondary caries in ECC. However, whether DMAEM@RA has an antimicrobial effect on *S. mutans* and *C. albicans* dual-species biofilms to prevent secondary caries in ECC has not yet been studied.

To simulate caries-active and caries-inactive states in vivo, in this study, we set four different cultural pH values (pH = 5, pH = 5.5, pH = 6, and pH = 7.4) for our experiments. The purpose of the study was to investigate the pH-responsive antimicrobial effect of DMAEM@RA on *S. mutans* and *C. albicans* dual-species biofilms, as well as to analyze the pH-responsive preventive effect of DMAEM@RA on secondary caries through the microbial demineralization model so as to explore whether the inhibitory effect of DMAEM@RA can only appear in an acidic environment.

Our hypotheses were as follows: (1) DMAEM@RA displayed a pH-responsive antimicrobial effect against *S. mutans* and *C. albicans* dual-species biofilms; (2) DMAEM@RA pH-responsively prevented the development of secondary caries by inhibiting the demineralization process and attenuating cariogenic virulence in *S. mutans* and *C. albicans* dual-species biofilms.

## 2. Results

### 2.1. DMAEM@RA Inhibited the Growth of S. mutans and C. albicans Dual-Species Biofilms in a pH-Dependent Manner

**Figure 1:**
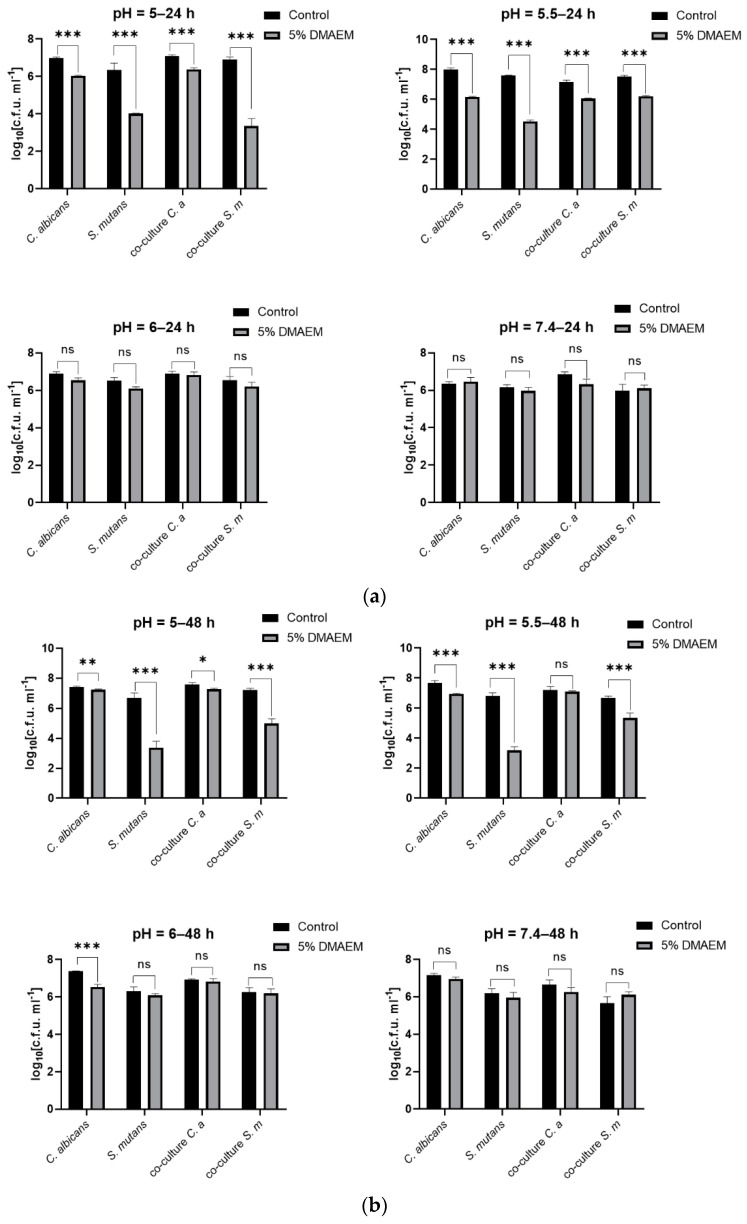
DMAEM@RA inhibited the growth of *S. mutans* and *C. albicans* dual-species biofilms in a pH-dependent manner. (**a**) CFU counts of the single biofilm and dual-species biofilms cultured for 24 h under different pH conditions. (**b**) CFU counts of the single biofilm and dual-species biofilms cultured for 48 h under different pH conditions. Data are presented as means ± standard deviation (*n* = 4, * *p* < 0.05, ** *p* < 0.01, and *** *p* < 0.001; ns, no statistical significance).

Firstly, we tested the antimicrobial effect of 5% DMAEM@RA against *S. mutans* and *C. albicans* dual-species biofilms activity under different pH conditions. As shown in Figure 1, the CFU results indicated that 5% DMAEM@RA could significantly inhibit the growth of dual-species biofilms cultured for 24 h (Figure 1a) and 48 h (Figure 1b) at pH 5 and 5.5 compared with the control groups, and therein, the amount of *S. mutans* decreased more significantly, with a >2 log10 reduction. At pH 6 and pH 7.4, no significant difference was found in the dual-species biofilms growth between the 5% DMAEM@RA groups and the control groups, with the 24 h or 48 h culturing times showing no effect. These results were consistent with the design of DMAEM@RA, which played an antimicrobial role only in acidic environments.

### 2.2. DMAEM@RA Played pH-Dependent Antibiofilm Roles on S. mutans and C. albicans Dual-Species Biofilms In Vitro

**Figure 2:**
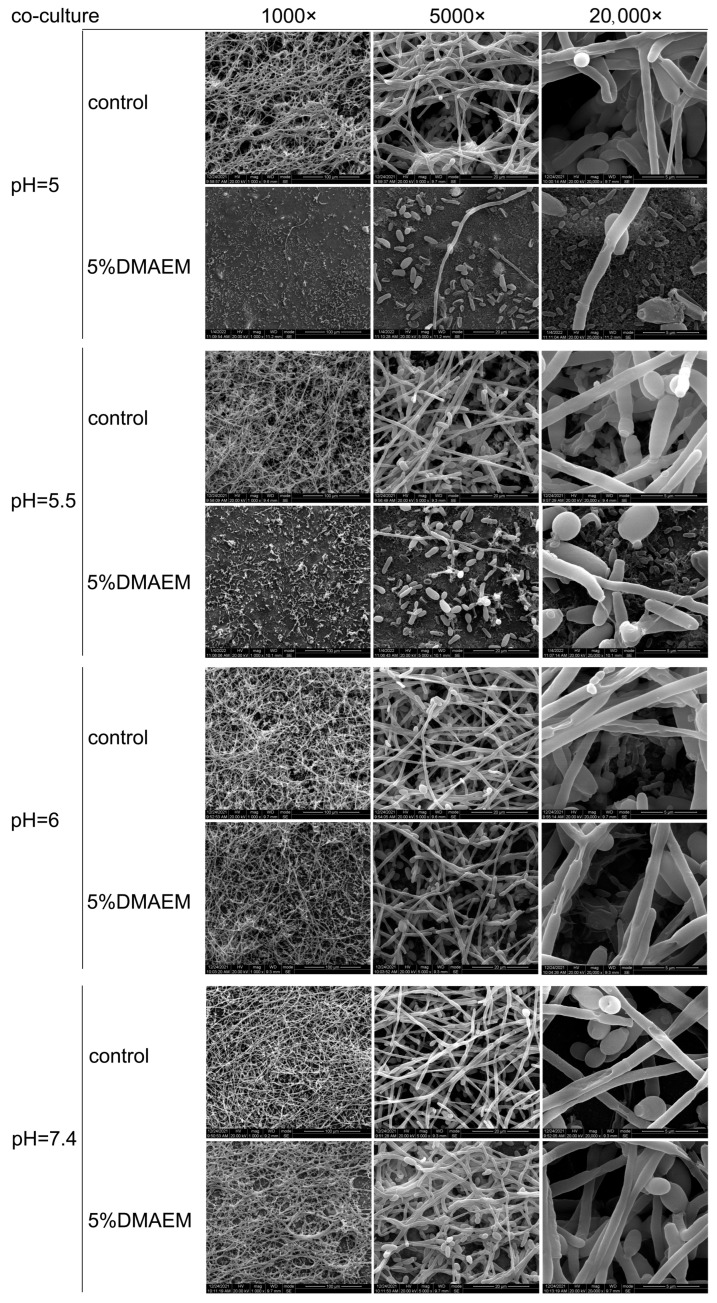
DMAEM@RA played pH-dependent antibiofilm roles on *S. mutans* and *C. albicans* dual-species biofilms in vitro. SEM images of *S. mutans* and *C. albicans* dual-species biofilms formed after 24 h in the 5% DMAEM@RA groups and the control groups at different pH values. The magnifications were 1000×, 5000×, and 20,000×, respectively.

SEM imaging showed that in contrast with the control groups, the 5% DMAEM@RA groups played significant antibiofilm roles on the *S. mutans* and *C. albicans* dual-species biofilms at pH 5 and 5.5 (Figure 2), and this phenomenon corresponded to the previous CFU results. While 5% DMAEM@RA had no obvious effect on the formation of biofilms compared with the controls at pH 6 and 7.4, in which condition the *C. albicans* mainly presented as a hypha, it served as the primary scaffold for *S. mutans* aggregation, proving the pH-dependent inhibitory effect of DMAEM@RA against the dual-species biofilms.

### 2.3. DMAEM@RA Decreased the Production of EPS of the S. mutans and Dual-Species Biofilms at pH 5 and 5.5

**Figure 3:**
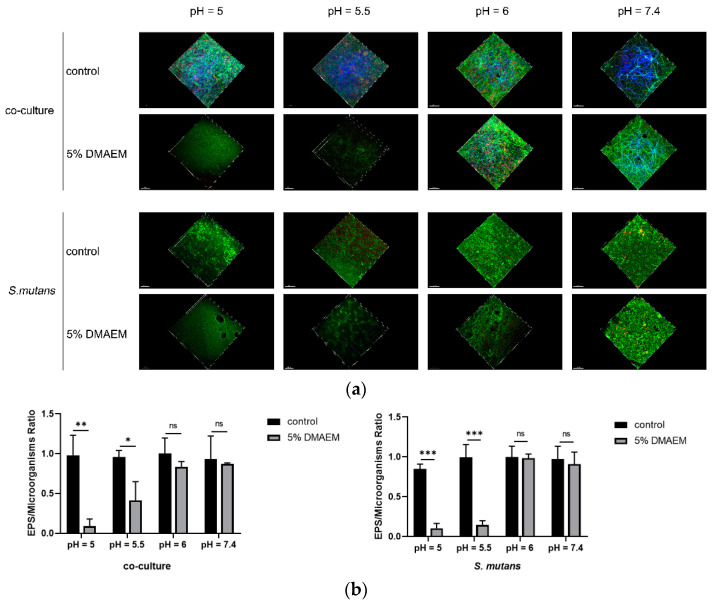
DMAEM@RA decreased the production of EPS of the *S. mutans* and dual-species biofilms at pH 5 and 5.5. (**a**) CLSM observation of single-*S. mutans* biofilm and dual-species biofilms at 24 h in different pH values groups. (**b**) Quantitative analysis of the ratios between the EPS and the microorganisms in the 5% DMAEM@RA groups and the control groups at different pH values. Data are presented as means ± standard deviation (*n* = 3, * *p* < 0.05, ** *p* < 0.01, and *** *p* < 0.001; ns, no statistical significance).

The three-dimensional microorganisms/extracellular polysaccharides staining images are shown in Figure 3a, from which we found that at pH 5 and 5.5, both the microorganisms and EPS of *S. mutans* and the co-culture biofilms were decreased remarkably under the stress of 5% DMAEM@RA. However, the dual-species biofilms grew vigorously and gathered closely in the control groups. Meanwhile, there were no significant differences in the EPS staining images between the 5% DMAEM@RA and the control groups when the pH was 6 and 7.4. These results were also consistent with the preceding CFU results, to some extent. Further, in line with the three-dimensional microorganisms/extracellular polysaccharides staining images, quantitative analysis also verified that the ratio between the EPS and the microorganisms in the 5% DMAEM@RA groups were significantly decreased in comparison with the control groups at pH 5 and 5.5 (Figure 3b). This kind of difference was not found at pH 6 and 7.4, illustrating that DMAEM@RA can effectively inhibit EPS production only in acidic environments.

### 2.4. DMAEM@RA pH-Responsively Reduced the Production of Lactic Acid in S. mutans and C. albicans Dual-Species Biofilms

**Figure 4:**
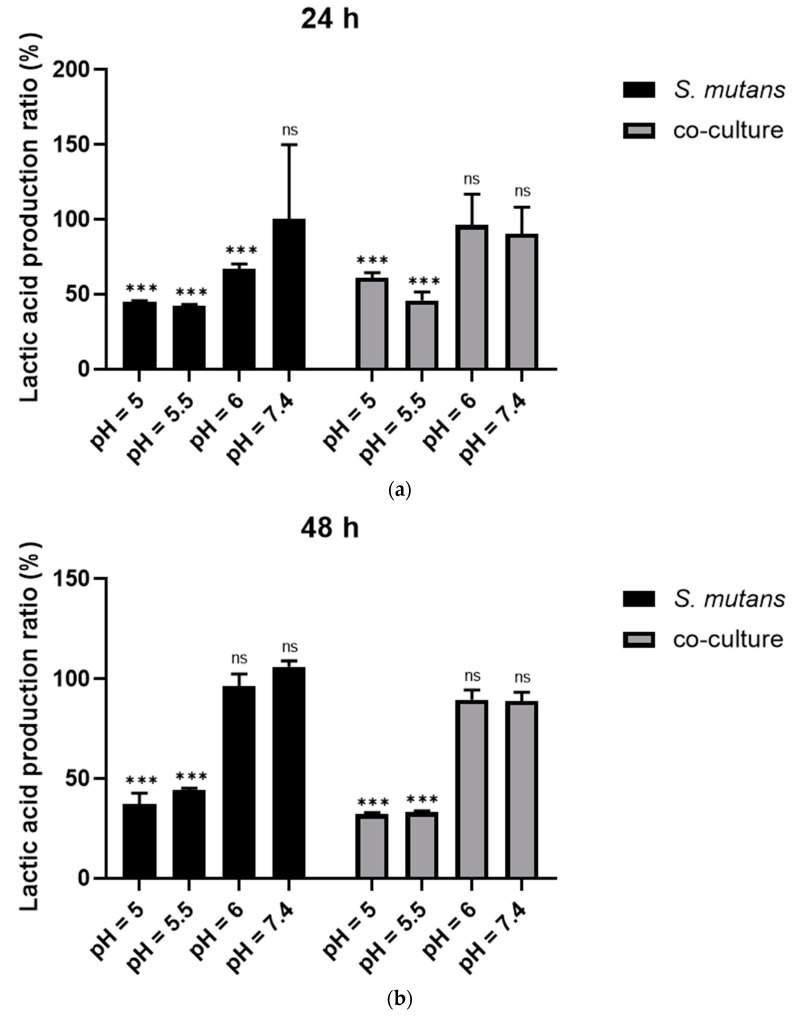
DMAEM@RA pH-responsively reduced the production of lactic acid in *S. mutans* and *C. albicans* dual-species biofilms. In single-*S. mutans* biofilm and dual-species biofilms: (**a**) the lactic acid production ratio of the DMAEM@RA group to the control group cultured for 24 h under different pH values; and (**b**) the lactic acid production ratio of the DMAEM@RA group to the control group cultured for 48 h at different pH values. Data are presented as means ± standard deviation (*n* = 5, *** *p* < 0.001; ns, no statistical significance).

The lactic acid production results indicated that compared to the control groups including 24 h *S. mutans* biofilm, as well as the dual-species biofilms of *S. mutans* and *C. albicans*, the samples that were cultured on 5% DMAEM@RA at pH 5 and 5.5 produced less lactic acid, which decreased by approximately 50% (Figure 4a). Similarly, the results indicated that the 5% DMAEM@RA also significantly reduced the acid production of the 48 h *S. mutans* biofilm and the dual-species biofilms at pH 5 and 5.5 (Figure 4b). However, no difference was observed in lactic acid production between the 5% DMAEM@RA group and the control groups at pH 6 and 7.4, confirming the pH responsiveness of DMAEM@RA when affecting acid production. The lactic acid production results were correlated with the CFU results, and the reduction of the CFU could partially affect the acid production of the DMAEM@RA groups at pH 5 and 5.5.

### 2.5. DMAEM@RA Inhibited Tooth Demineralization Caused by S. mutans and C. albicans Dual-Species Biofilms in a pH-Responsive Way In Vitro

**Figure 5:**
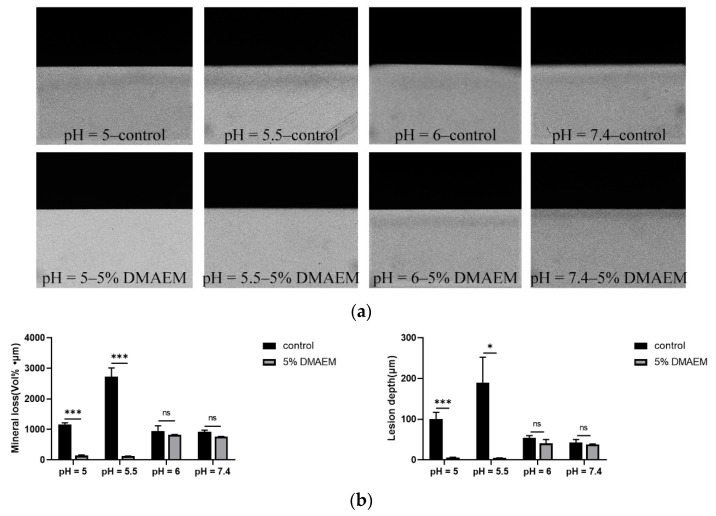
DMAEM@RA inhibited the tooth demineralization caused by *S. mutans* and *C. albicans* dual-species biofilms in a pH-responsive way in vitro. (**a**) TMR microradiographs of different groups after treatment for 3 days. (**b**) The values of mineral loss and lesion depth of different groups after treatment for 3 days. Data are presented as means ± standard deviation (*n* = 4, * *p* < 0.05 and *** *p* < 0.001; ns, no statistical significance).

TMR was applied to further investigate if 5% DMAEM@RA could prevent the tooth demineralization caused by *S. mutans* and *C. albicans* dual-species biofilms. The TMR microradiographs are shown in Figure 5a. The demineralization degree of the pH = 5–5% DMAEM@RA group and the pH = 5.5–5% DMAEM@RA group was much lower than that of the corresponding control groups, which illustrated that 5% DMAEM@RA could affect enamel demineralization only at pH 5 and 5.5, rather than at pH 6 and 7.4. At the same time, the mineral loss and lesion depth results in Figure 5b illustrated that 5% DMAEM@RA significantly decreased the demineralization degree of enamel caused by the dual-species biofilms in the microbial demineralization model in vitro at pH 5 and 5.5. At these two pH conditions, the mineral loss of the 5% DMAEM@RA groups were evidently lower than the control groups, and the lesion depths of the 5% DMAEM@RA groups were also shallower than control groups. However, these kinds of phenomena were not seen at pH 6 and 7.4. These results again confirmed that pH-responsive DMAEM@RA could only inhibit demineralization under acidic environments.

### 2.6. DMAEM@RA Changed the Expression of the Cariogenic Genes, Virulence-Associated Genes, and pH-Regulated Genes of the Dual-Species Biofilms at pH 5 and 5.5

**Figure 6:**
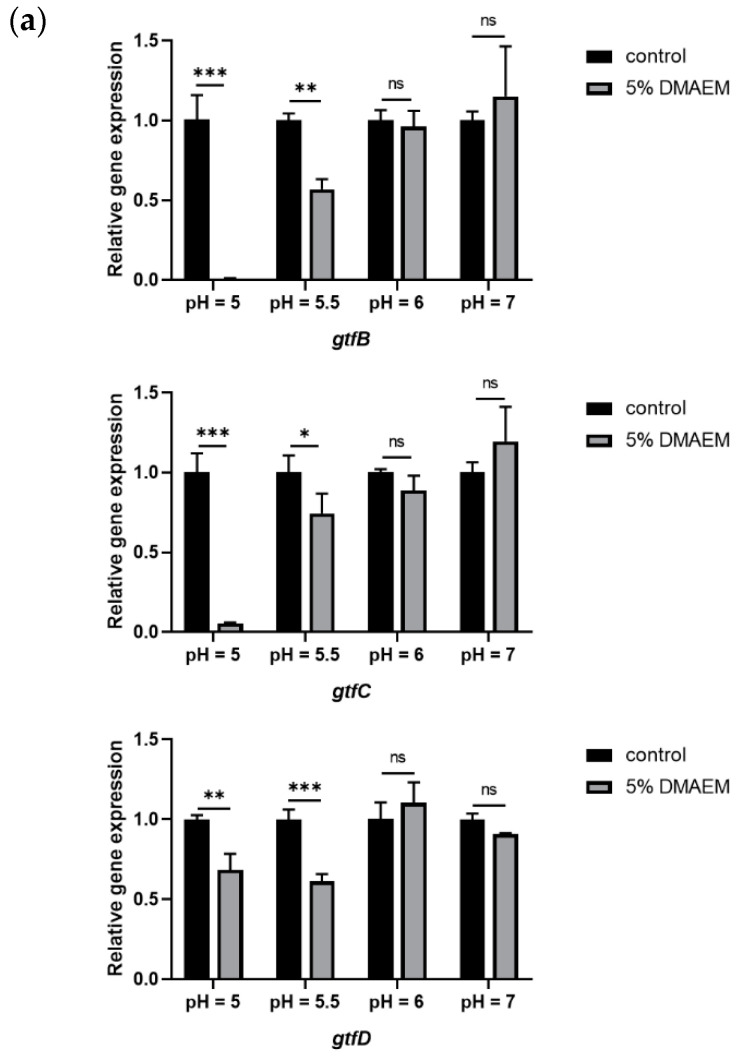

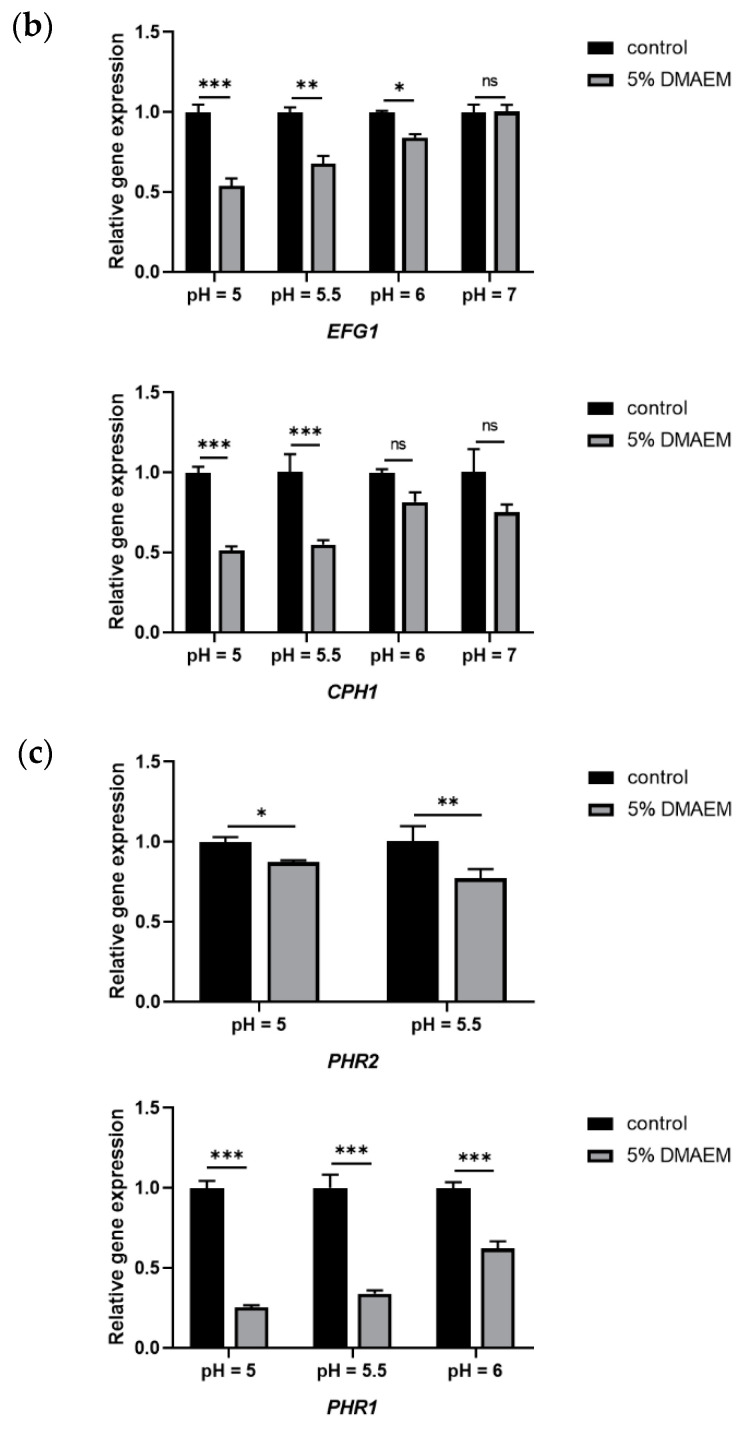
DMAEM@RA changed the expression of cariogenic genes and virulence-associated genes as well as the pH-regulated genes, of dual-species biofilms at pH 5 and 5.5. (**a**) The expression of *gtf*-associated genes (*gtfB*, *gtfC*, and *gtfD*) of the different treatment and pH groups. (**b**) The differential expression of the virulence-associated genes of *C. albicans* (*EFG1* and *CPH1*) in the 5% DMAEM@RA groups and the control groups at different pH values. (**c**) The effect of 5% DMAEM@RA on the expression of the critical pH-regulated genes of *C. albicans* (*PHR1* and *PHR2)* at different pH values. Data are presented as means ± standard deviation (*n* = 4, * *p* < 0.05, ** *p* < 0.01, and *** *p* < 0.001; ns, no statistical significance).

To verify the effect of 5% DMAEM@RA on a dual-species gene level, we selected genes related to the cariogenic virulence of *S. mutans*, including *gtfB*, *gtfC*, and *gtfD*, to detect the anti-caries effect of 5% DMAEM@RA at different pH values in dual-species biofilms. Meanwhile, the virulence-associated genes (*EFG1* and *CPH1*) and pH-related genes (*PHR1* and *PHR2*) of *C. albicans* were also tested to explore the pH-responsive anti-*C. albicans* effect of 5% DMAEM@RA in dual-species biofilms. The experimental results demonstrated that compared with the control groups, 5% DMAEM@RA markedly downregulated the expression of *gtf*-associated genes at pH 5 and 5.5, among which the expression of *gtfB* decreased most obviously (Figure 6a). Moreover, as shown in Figure 6b, the expression of the virulence-associated genes (*EFG1* and *CPH1*) of *C. albicans* reflected similar results: 5% DMAEM@RA significantly inhibited the expression of *EFG1* and *CPH1* at pH 5 and 5.5, and the expression of these two genes decreased by almost 50%. In contrast, the expression of *gtfB*, *gtfC*, *gtfD*, and *CPH1* between the experimental groups and the control groups at pH 6 and 7.4 had no significant difference, and only the expression of *EFG1* showed a small decrease at pH 6. *PHR1* and *PHR2* are crucial factors determining the environmental adaptability of *C. albicans* under different pH environments [29]. *PHR1* is expressed at pH 5.5 or higher, and *PHR2* is expressed at pH 5.5 or lower [30]. We further analyzed the expression of these two critical pH-regulated genes under the treatment of 5% DMAEM@RA at different pH values. Interestingly, although the results showed that the expression of *PHR2* was downregulated in the 5% DMAEM@RA groups when cultured at pH 5 and 5.5, which agreed with our expectations, surprisingly, we found that at pH 6 and 7.4, the expression of *PHR1* was downregulated in the DMAEM@RA groups (Figure 6c).

## 3. Discussion

Resin composite restoration is currently the most widely used clinical treatment for ECC [9]. However, secondary caries are still a major cause of dental restoration failure, especially for young children due to their poor oral hygiene maintenance and other reasons [11]. 

At present, both *S. mutans* and *C. albicans* have been proved to be significant pathogens related to ECC, and the pH decrease caused by the acid production of dual-species biofilms is one of the main reasons for the secondary caries of ECC [31,32,33]. Further, the defects of restorative materials themselves, such as the lack of an antimicrobial effect in acidic environments, are more likely to cause secondary caries in children.

Therefore, because of their special tooth-restoration interface connector location, a pH-responsive adhesive system is an important means to prevent secondary caries. In our previous study, DMAEM@RA was proven to exhibit pH-responsive antibacterial activity [28]. Therefore, we further analyzed the pH-sensitive antimicrobial effect of DMAEM@RA on *S. mutans* and *C. albicans* dual-species biofilms, and whether it could have a positive effect on preventing secondary caries in different pH environments in children with ECC. 

Firstly, we investigated the antimicrobial effect of DMAEM@RA. The results revealed that DMAEM@RA could significantly inhibit the growth of dual-species biofilms at pH 5.5, which is exactly the critical pH value for the occurrence and development of dental caries. With the growth inhibition of dual-species biofilms by DMAEM@RA, the production of lactic acid and EPS was further reduced.

EPS is a crucial virulence factor of cariogenic biofilms, and many studies have proven that EPS plays a vital role in the interactions of *S. mutans* and *C. albicans* and that the existence of *C. albicans* increases the production of the EPS matrix, and further, that GtfB derived from *S. mutans* can quickly bind to the cell surface of *C. albicans* via fungal mannan and β-glucan [31,34,35] and promote the generation of an extensive extracellular matrix, resulting in the highly virulent mixed biofilms of severe childhood caries [36,37]. Therefore, regulating the EPS production of dual-species biofilms is very important in preventing dental caries and secondary caries in young children. Accordingly, the confocal laser scanning microscope examinations showed that the EPS of *S. mutans* and co-culture biofilms were decreased remarkably after exposure to DMAEM@RA in acidic environments. This means that DMAEM@RA can effectively reduce the synthesis of EPS at a cariogenic pH, thereby inhibiting the progression of caries.

Acid production is one of the major processes in caries formation. A previous study has discovered that lactic acid produced by *S. mutans* could be used as a substrate by *C. albicans* [14]. Yang et al. also demonstrated that the *S. mutans* antigen I/II mediated the increase in the acid production of *C. albicans* in dual-species biofilms [38]. Therefore, reducing the metabolic activity, decreasing the acid production, and inhibiting the demineralization ability of dual-species biofilms are also very important in preventing dental caries and secondary caries in young children. According to the results of the lactic acid measurements, DMAEM@RA significantly reduced the acid production at 24 h, as well as at 48 h, of *S. mutans* biofilms and dual-species biofilms at pH 5 and 5.5. These data indicated that DMAEM@RA inhibited the acid production potential of *S. mutans* and *C. albicans* dual-species biofilms and directly impaired their cariogenic capacity. Further, by performing TMR analysis, we found that DMAEM@RA, in vitro, inhibited the tooth demineralization caused by dual-species biofilms in a pH-responsive manner, and DMAEM@RA significantly decreased enamel mineral loss and demineralized lesion depth at pH 5 and 5.5. Taken together, these results demonstrate that DMAEM@RA shows great potential for inhibiting the demineralization caused by dual-species biofilms in acidic environments, thus preventing secondary caries in young children.

We further investigated whether DMAEM@RA induced a variation of the virulence expression of the dual-species biofilms. Glucosyltransferases consisting of GtfB, C, and D are considered to be key contributors to cariogenic plaque formation [39]. GtfB can quickly bind to the cell surface of *C. albicans* and promote the generation of an extensive extracellular matrix, resulting in the highly cariogenic biofilm of severe childhood caries [35]. Therefore, targeting the inhibition of GTF in dual-species biofilms is very important for preventing secondary caries. Our qPCR results demonstrated that compared with controls, DMAEM@RA evidently downregulated the expression of *gtf*-associated genes at pH 5 and 5.5, among which the expression of *gtfB* decreased most obviously, suggesting that DMAEM@RA effectively inhibited the cariogenicity of dual-species biofilms when the local environment was acidic. *EFG1* is one of the key transcription factors that regulates *C. albicans* biofilms and the EPS matrix formation in *S. mutans* and *C. albicans* dual-species biofilms [31,40,41,42]. The *CPH1* of *C. albicans* is another key regulator in dual-species biofilms as it induces the exposure of β (1,3)-glucan, which is the exact the binding site for GtfB derived from *S. mutans* [43,44]. Therefore, inhibiting the expression of *EFG1* and *CPH1* may reduce the cariogenicity of dual-species biofilms, to a certain extent. Our results showed that the expression of *EFG1* and *CPH1* in the DMAEM@RA groups was significantly downregulated at pH 5 and 5.5, illustrating that DMAEM@RA may effectively weaken the virulence of *C. albicans* in dual-species biofilms. Finally, the PHR1 and PHR2 proteins belong to the GH72 family of β-(1,3)- glucanosyltransferases and play a vital role in the cell wall assembly, as well as the adaptive response to pH changes, of *C. albicans* [45]. In the context of bacterium–fungus interactions, these genes are also considered to be related to virulent biofilm formation [37]. Further, it was discovered that the absence of pH-related genes, especially *PHR2*, which is related to the acid adaptation of *C. albicans*, can lead to a significant decrease in the fungal cells of cariogenic biofilms. Correspondingly, our results showed that the application of DMAEM@RA significantly downregulated the expression of PHR2 when the dual-species biofilms were cultured at pH 5 and 5.5, suggesting that in a cariogenic environment, DMAEM@RA can reduce the cariogenic virulence of dual-species biofilms, to a certain extent. 

In conclusion, DMAEM@RA exhibited a pH-responsive antimicrobial effect against *S. mutans* and *C. albicans* dual-species biofilms by inhibiting microbial growth and EPS production in an acidic environment. Moreover, DMAEM@RA can pH-responsively inhibit the demineralization process and attenuate the cariogenic virulence of dual-species biofilms. Therefore, a novel, intelligent, pH-responsive anti-caries resin adhesive containing DMAEM could have great potential for future clinical applications in preventing secondary caries, especially in children with ECC. However, the present study has certain limitations, and more in-depth studies are needed to understand the specific antimicrobial and caries prevention mechanisms of DMAEM@RA. More in vivo evidence is also needed for the future clinical application of DMAEM@RA.

## 4. Materials and Methods

### 4.1. Preparation of DMAEM@RA

As in our previous study, DMAEM was incorporated into the dental adhesive resin Clearfil SE Bond (Kuraray Dental, Tokyo, Japan) with the mass fraction of 5%, yielding DMAEM@RA, following the method of TA@RAs-modified dental adhesive resin [28]. Dental adhesives containing 5% DMAEM@RA did not affect the bonding strength and biocompatibility of the adhesives [28]. Then, we used a magnetic stirrer to stir the mixture continuously overnight. Meanwhile, the Clearfil SE Bond was used as the control group. Resin disks were prepared based on previous studies [46]. A 48-well plate cover (Costar, Corning Inc., Corning, NY, USA) was used as a mold for preparing the resin disks. After that, we applied approximately 20 μL of bonding agents to the surface of the resin disks, which were then cured for 15 s with an LED curing light (LED. B, Woodpecker, Guilin, China) [47]. Then, we immersed the samples in deionized water for 24 h to remove the unpolymized monomers. The resin disks were sterilized using ethylene oxide in an ethylene oxide sterilizer (Anprolene AN 74i, Andersen, Haw River, NC, USA).

### 4.2. Bacterial Strains and Growth Conditions

*S. mutans* UA159 and *C. albicans* SC5314 were provided by the State Key Laboratory of Oral Diseases, Sichuan University (Chengdu, China). *S. mutans* UA159 was anaerobically cultured in a brain–heart infusion broth (BHI) (85% N_2_, 10% H_2_, and 5% CO_2_, 37 °C). *C. albicans* SC5314 was aerobically grown in yeast extract peptone dextrose medium (YPD) (35 °C). The UA159 and SC5314 were cultured overnight and resuspended in BHI and YPD, respectively, then grown to the logarithmic growth period. After that, the bacterial cells and yeast were centrifuged and resuspended in a YNBB medium (0.67% YNB, 2.5 mM *N*-acetylglucosamine, 75 mM Na2HPO4-NaH2PO4, 0.5% sucrose, and 0.2% casamino acids) [48], and the inoculum for the dual species was adjusted to 2 × 10^6^ CFU/mL for *S. mutans* and 2 × 10^4^ CFU/mL for *C. albicans* [44]. At the same time, di-Sodium hydrogen phosphate (Sigma-Aldrich, Shanghai, China) and a citrate (Sigma-Aldrich, Shanghai) buffer system were used to adjust and maintain the different pH values (5, 5.5, 6, and 7.4) of the YNBB medium.

### 4.3. Colony-Forming Units (CFU) Counts

The number of viable microorganisms in the dual-species biofilms of *S. mutans* and *C. albicans* treated with DMAEM@RA was evaluated by CFU counting. The 24 h and 48 h biofilms that formed on the resin disks at different pH cultures were washed twice with phosphate buffered saline (PBS). The biofilms were then scraped with a sterilizing blade and ultrasound/eddy currents were performed in PBS [49]. The microorganism viability was assessed by using BHI agar plates after serial dilution in PBS. 

### 4.4. Scanning Electron Microscope (SEM) Examination

The structures of the *S. mutans* and *C. albicans* dual-species biofilms were observed using SEM. For biofilm formation, *S. mutans*, with an initial concentration of 2 × 10^6^ CFU/mL, and *C. albicans*, with an initial concentration of 2 × 10^4^ CFU/mL, were co-cultured in YNBB with different pH values. After 24 h, the biofilms that formed on the resin disks of the 24-well plates were washed twice with PBS and fixed overnight with 2.5% glutaraldehyde [50]. Then, the samples underwent dehydration of gradient ethanol (50%, 60%, 70%, 80%, 90%, 95%, and 100%) and were examined by scanning electron microscopy (Quanta 200, FEI, Hillsboro, OR, USA) at 1000×, 5000×, and 20,000× magnifications.

### 4.5. Exopolysaccharide (EPS) Staining

The EPS production of the dual-species biofilms formed by *S. mutans* and *C. albicans* was detected by confocal laser scanning microscope examinations. Alexa Fluor 647 (Thermo Fisher Scientific, Waltham, MA, USA) was supplemented into the different pH culture media, with a final concentration of 1 μL/mL at the beginning of culturing the biofilms. After 24 h, the suspensions were sucked up and the biofilms were gently washed with sterile deionized water. The biofilms that formed on the resin disks in the 24-well plates were firstly stained with 2.5 μM SYTO 9 nucleic acid stain (Thermo Fisher Scientific, USA) for 15 min at an ambient temperature and away from light. Then, we removed the SYTO 9 dye solution and rinsed the residual dye solution gently with sterile deionized water. After that, we added Calcoflour White Stain (Sigma-Aldrich, St. Louis, MO, USA), with a final concentration of 10 μg/mL, and stained the biofilms for 1 min in the dark. The exopolysaccharides were labeled with Alexa Fluor 647, which fluoresced red, the microbe cells were labeled with SYTO 9 green-fluorescent nucleic acid stain, and the *C. albicans* cultures were labeled with Calcoflour White Stain, which fluoresced blue [51,52]. The staining images were taken with a confocal laser scanning microscope (Olympus FV1000, Tokyo, Japan), using Imaris 7.4.2 (Bitplane, Zürich, Switzerland) to reconstruct the three-dimensional structure of the biofilms, and we calculated the ratios between the EPS and the microorganisms using Image-Pro-Plus 6.0 (Media Cybernetics; Bethesda, MD, USA) and Matrix Laboratory (Mathworks, Natick, MA, USA).

### 4.6. Lactic Acid Measurement

Resin disks with the 24 h and 48 h biofilms cultured at different pH values were rinsed with cysteine peptone water (Sigma-Aldrich, Saint Louis, MO, USA) and then transferred to new 24-well plates containing buffered peptone water with 0.2% sucrose (S8270; Solarbio, Beijing, China). After that, the whole system was incubated for 3 h at 5% CO_2_, at 37 °C, and the production of lactic acid was monitored with a microplate reader (SPECTROstar Nano; Gene, Hong Kong, China) at OD_340_ nm [52,53]. The standard curves were prepared using a lactic acid standard (Supelco Analytical; Bellefonte, PA, USA). Then, the lactic acid production ratios of the DMAEM@RA groups to the control groups were calculated.

### 4.7. Transverse Microradiography (TMR) Analysis

We investigated the anticaries effect of DMAEM@RA using the microbial demineralization model, and the mineral loss and caries lesion depth were measured by TMR. Briefly, the labial dental crowns of extracted bovine teeth with no damage, cracks, or fluorine spots were cut into enamel blocks (5 mm × 5 mm × 4 mm). The tooth specimens were applied by primer and DMAEM@RA or Clearfil SE Bond and then light-cured. The composite resin was grafted to the binding surface, making the specimens approximately 10 mm in length [28]. The rest of sides of the blocks were covered, except the side used for biofilm incubation, and we disinfected the samples with ethylene oxide. We cultured the dual-species biofilms in a YNBB medium at different pH values and refreshed the medium every 24 h. Then, we rinsed the samples with PBS to remove the biofilms after treating them with the microbial demineralization treatment for 3 d (5% CO_2_, 37 °C). Then, the segments were cut and polished to a thickness of approximately 100 ± 10 μm [54]. The enamel bonding interface was analyzed by transverse microradiography. We took slices with an aluminum calibration step wedge using a monochromatic CuK X-ray source (Philips B.V., Amsterdam, The Netherlands). The transmitted light microscope (Axioplan; Zeiss, Oberkochen, Germany) was used to analyze the film. Transversal Microradiography Software 2006 (Inspektor Research Systems BV, Amsterdam, Netherlands) was used to analyze the lesion depth and mineral loss [55].

### 4.8. RNA Extraction and Quantitative Real-Time PCR (qPCR)

We used qPCR to investigate the different expressions of the cariogenic genes (*gtfB*, *gtfC*, and *gtfD*), virulence-associated genes (*EFG1* and *CPH1*), and pH-regulated genes (*PHR1*/*PHR2*) of the dual-species biofilms between the DMAEM@RA and the control groups. The dual-species biofilms were cultured in YNBB with different pH values for 6 h (5% CO_2_, 37 °C), then the 6 h biofilms that formed on the resin disks of the different groups in 24-well plates were collected by centrifugation. The total RNA was extracted with a MasterPure™ Complete DNA and RNA Purification Kit (lucigen, Middleton, WI, USA) and the RNA concentration and purity were determined using NanoDrop™ One (Thermo Fisher Scientific, USA) [56]. Then, we synthesized the complementary DNA with a PrimeScript RT Reagent Kit (TaKaRa, Tokyo, Japan). The qPCR was performed via QuantStudio 6 and finished with a Hieff qPCR SYBR Green Master Mix kit (Yeasen Biotechnology, Shanghai, China), following its two-step amplification procedure as follows: pre-incubation at 95 °C for 5 min; amplification for 40 cycles, including denaturation at 95 °C for 10 s; and annealing/extension at 60 °C for 30 s. *S. mutans* UA159 16S rRNA and *C. albicans* SC5314 18S rRNA were selected as the internal controls. The specific forward and reverse primers were synthesized at TsingKe biotechnology, China, and are listed in Table 1 [44,57,58]. The gene expression was normalized with the reference gene 16S or 18S, respectively, using the 2^−ΔΔ^CT method.

### 4.9. Statistical Analysis

One-way analysis of variance (ANOVA) was used to investigate the significant effects of the variables, followed by the Student–Newman–Keuls test. Tukey’s multiple-comparison test was applied to compare the data in each group. The *t*-test was performed for two groups. Statistical analysis of the data was performed with SPSS, version 22.0 (SPSS; Chicago, IL, USA) at a significance level (*p* value) of 0.05. All experiments were repeated at least three times independently.

## Figures and Tables

**Table 1:** Specific primers used for qPCR.

Gene	Sequence (5′→3′)	Template Strand
16S rRNA	F R	AGCGTTGTCCGGATTTATTG CTACGCATTTCACCGCTACA
*gtfB*	F R	CACTATCGGCGGTTACGAAT CAATTTGGAGCAAGTCAGCA
*gtfC*	F R	GATGCTGCAAACTTCGAACA TATTGACGCTGCGTTTCTTG
*gtfD*	F	TTGACGGTGTTCGTGTTGAT
	R	AAAGCGATAGGCGCAGTTTA
18S rRNA	F	TCTTTCTTGATTTTGTGGGTGG
	R	TCGATAGTCCCTCTAAGAAGTG
*EFG1*	F	ACGTGGTAGAAGAGATGGGA
	R	TGCATTAGGAGTTACTCCGG
*CPH1*	F	GGTGGCGGCAGTGATAGTG
	R	GTGTACTCCGGTGACGATTTTTC
*PHR1*	F	GGTTTGGTTCTGGTTGATGG
	R	AGCAGCAGTTCCTGGACATT
*PHR2*	F	CTCCTCCATTTCCAGAACCA
	R	CGTCTGAATCAACCTTGTCG

## Data Availability

Not applicable.
